# Supplementary material for: A physicochemical perspective of aging from single-cell analysis of pH, macromolecular and organellar crowding in yeast
Source: eLife. 2020 Sep 29;9:e54707. doi: 10.7554/eLife.54707 (PMC7556870; doi:10.7554/eLife.54707)
Supplement: Supplementary file 1. [file elife-54707-supp1.docx]

**Table S1.** Strains used in this study.

| Strain name | Relevant Genotype | Source |
| --- | --- | --- |
| SMY8, TEF1-CrGE-NLS | *MATa leu2Δ0 met15Δ0 ura3Δ, his3Δ1::P_TEF1_-his_6_crGENLS-HIS3* | This study |
| SMY12, TEF1-pHluorin | *MATa leu2Δ0 met15Δ0, ura3Δ0 his3Δ1::P_TEF1_-pHluorin-HIS3* | This study |
| SMY15, TEF1-CrGE2.3 | *MATa leu2Δ0 met15Δ0 ura3Δ0 his3Δ1::P_TEF1_-his_6_crGE2.3-HIS3* | This study |
| Sequence CrGE2.3: | MGHHHHHHKGEELFTGVVPILVELDGDVNGHKFSVSGEGEGDATYGKLTLKFICTTGKLPVPWPTLVTTLTYGVQCFSRYPDHMKQHDFFKSAMPEGYVQERTIFFKDDGNYKTRAEVKFEGDTLVNRIELKGIDFKEDGNILGHKLEYNYNSHNVYIMADKQKNGIKVNFKIRHNIEDGSVQLADHYQQNTPIGDGPVLLPDNHYLSTQSKLSKDPNEKRDHMVLLEFVTAAGITLGMDELYKGSGGSGGSGGSGGSGGSGAEAAAKEAAAKEAAAKEAAAKEAAAKEAAAKAGSGGSGGSGGSGGSGGSGAEAAAKEAAAKEAAAKEAAAKEAAAKEAAAKAGSGGSGGSGGSGGSGGSMVSKGEAVIKEFMRFKVHMEGSMNGHEFEIEGEGEGRPYEGTQTAKLKVTKGGPLPFSWDILSPQFMYGSRAFIKHPADIPDYYKQSFPEGFKWERVMNFEDGGAVTVTQDTSLEDGTLIYKVKLRGTNFPPDGPVMQKKTMGWEASTERLYPEDGVLKGDIKMALRLKDGGRYLADFKTTYKAKKPVQMPGAYNVDRKLDITSHNEDYTVVEQYERSEGRHSTGGMDELYK- | |
