# Supplementary material for: A physicochemical perspective of aging from single-cell analysis of pH, macromolecular and organellar crowding in yeast
Source: eLife. 2020 Sep 29;9:e54707. doi: 10.7554/eLife.54707 (PMC7556870; doi:10.7554/eLife.54707)
Supplement: Supplementary file 2. [file elife-54707-supp2.docx]

**Table S2.** Primer sequences used in this study.

| Primer name | Sequence |
| --- | --- |
| F1_SM | CGAGCTACTAGTCATAGCTTCAAAATGTTTCTACTCC |
| R1_SM | GCAGTCTAGATTACTTGTATAATTCATCCATACC |
| F2_SM | ATGCAAGCTTGGAAAAAAATGAGTAAAGGAGAAGAAC |
| R2_SM | GCCCTCTAGACCCGGGTTATTTGTATAGTTCATCCATGCC |
| F3_SM | AGCCATGCCAAGCTTATCG |
| R3_SM | ACGCTCTAGATTACTTGTACAGCTCGTCCATGC |
| F4_SM | TTGAAGGAAGGCCGTCAAGG |
| R4_SM | ACGCTCTAGACCCGGGTTATTTGTACAGCTCATCC |
