# Supplementary material for: A physicochemical perspective of aging from single-cell analysis of pH, macromolecular and organellar crowding in yeast
Source: eLife. 2020 Sep 29;9:e54707. doi: 10.7554/eLife.54707 (PMC7556870; doi:10.7554/eLife.54707)
Supplement: Supplementary file 3. [file elife-54707-supp3.docx]

**Table S3**. Comparison between measuring cytoplasmic acidity in aging every 10 hours (Figure 1*C,D,F,* Figure 1-Figure supplement 1) or every hour (Figure 1*E*, Figure1-Figure supplement 2).

| Dataset  time resolution | Average lifespan  at 20 h  (divisions) | Average lifespan  at 30 h (divisions) | Number of cells | Frequency of imaging | Duration  of the experiment |
| --- | --- | --- | --- | --- | --- |
| Low | 16  SD: 3.08 | 20  SD: 4.89 | 80 | Every 10 h | 80 h |
| High | 13  SD: 2.93 | 17  SD: 4.75 | 50 | Every 1 h | 50 h |
